# Progress towards an inducible, replication-proficient transposon delivery vector for *Chlamydia trachomatis*

**DOI:** 10.12688/wellcomeopenres.16665.1

**Published:** 2021-04-13

**Authors:** Rachel J. Skilton, Colette O'Neill, Nicholas R. Thomson, David J. Lampe, Ian N. Clarke

**Affiliations:** 1 Molecular Microbiology Group, Faculty of Medicine, University of Southampton, Southampton, Hants, SO16 6YD, UK; 2 Wellcome Trust Sanger Institute, Wellcome Trust Genome Campus, Hinxton, Cambs, CB10 1RQ, UK; 3 London School of Hygiene and Tropical Medicine, London School of Hygiene and Tropical Medicine, London, WC1E 7HT, UK; 4 Department of Biological Sciences, Duquesne University, 600 Forbes Ave., Pittsburgh, Pennsylvania, 15116, USA

**Keywords:** transposon, Chlamydia, transformation, Himar1

## Abstract

**Background**

Genetic systems have been developed for *Chlamydia* but the extremely low transformation frequency remains a significant bottleneck.  Our goal is to develop a self-replicating transposon delivery vector for *C. trachomatis* which can be expanded prior to transposase induction.

**Methods**

We made *E. coli*/ *C. trachomatis* shuttle vectors bearing the *Himar1* C9  transposase under control of the *tet* promoter and a novel rearrangement of the *Himar1* transposon with the β-lactamase gene.  Activity of the transposase was monitored by immunoblot and by DNA sequencing.

**Results**

We constructed pSW2-mCh-C9, a *C. trachomatis* plasmid designed to act as a self-replicating vector carrying both the *Himar1* C9  transposase under *tet* promoter control and its transposon.  However, we were unable to recover this plasmid in *C. trachomatis* following multiple attempts at transformation.

Therefore, we assembled two new deletion plasmids pSW2-mCh-C9-ΔTpon carrying only the *Himar1* C9  transposase (under *tet* promoter control) and a sister vector (same sequence backbone) pSW2-mCh-C9-ΔTpase carrying its cognate transposon.  We demonstrated that the biological components that make up both pSW2-mCh-C9-ΔTpon and pSW2-mCh-C9-ΔTpase are active in *E. coli.  *Both these plasmids could be independently recovered in *C. trachomatis.*

We attempted to perform lateral gene transfer by transformation and mixed infection with *C. trachomatis *strains bearing **pSW2-mCh-C9-ΔTpon and pSW2-RSGFP-Tpon **(a green fluorescent version of **pSW2-mCh-C9-ΔTpase).  Despite success in achieving mixed infections, it was not possible to recover progeny bearing both versions of these plasmids.

**Conclusions**

We have designed a self-replicating plasmid vector pSW2-mCh-C9 for *C. trachomatis* carrying the *Himar1* C9  transposase under *tet* promoter control.  Whilst this can be transformed into *E. coli* it cannot be recovered in *C. trachomatis. * Based on selected deletions and phenotypic analyses we conclude that low level expression from the *tet* inducible promoter is responsible for premature transposition and hence plasmid loss early on in the transformation process.

## Introduction

*Chlamydia trachomatis* is a major human pathogen, causing trachoma, the most prevalent infectious blinding disease ^1^, and is also the main bacterial agent of sexually transmitted infections worldwide ^2^. *C. trachomatis* is an obligate intracellular pathogen with a unique developmental cycle which involves two distinct forms ^3^. The extracellular infectious form is known as an elementary body (EB). The EB attaches to a susceptible eukaryotic host cell and is then taken up within a membrane structure known as an inclusion ^4^. The early chlamydial inclusion evades phagolysosomal fusion as the EBs secrete and synthesise proteins that modify the inclusion membrane subverting the normal cellular vesicle pathway. Once an EB has been taken up within a nascent inclusion it will, over several hours (the process takes 8–12 hours depending on strain and host cell and culture conditions) differentiate into a reticulate body (RB). RBs are the non-infectious form of the microorganism and these then follow a normal bacterial growth curve dividing by binary fission for 8 to 10 generations ^5^. During the exponential growth phase of *C. trachomatis* L2 the RBs multiply at a rate of one bacterial division/chromosomal replication every 4 to 6 hours and during the process most asynchronously differentiate back to become EBs. Once the inclusion reaches a critical late phase of development, the host cell lyses. At this point of maturation the infected cell contains around 500 – 1000 EBs which are released to infect further cells and commence a new developmental cycle ^5^.

Progress in studying the molecular biology and genetics of *Chlamydia* has been severely hampered because it is not possible to unravel the stages of the intracellular developmental cycle from its close co-ordination with the host. There remains no routine cell-free culture system, nor systems to allow arrest/recommencement of the developmental cycle, although cell-free extracts have been described that support RB metabolism, but not replication ^6^. Thus the ability to analyse and purify components of chlamydial development lags far behind what is possible with the free-living bacteria that can be cultivated on defined media. Ten years ago a robust plasmid-based transformation system was developed in our laboratory by inducing bacterial competence with CaCl _2 _and using the endogenous chlamydial plasmid as a part of an *E. coli* shuttle/cloning vector ^7^. Since then a great deal of progress has been made in studying chlamydial genetics ^8^. It is now possible to make targeted insertions and gene deletions in the chlamydial chromosome. However, a very signficant difficulty remains - the extremely low transformation frequency of *Chlamydia*. This makes chlamydial genetics both time consuming and technically demanding, especially so for mutagenesis, since overall gene essentiality is unknown and thus in the absence of any biological data target genes chosen for study could be impossible to ‘knock out’. This problem is further compounded as it is not possible to provide conditionally-controlled complementary copies of genes on a separate vector whilst making knock outs of ‘essential’ genes since there is only type one delivery vector (they are all based on the endogenous plasmid) and different variants of the same chlamydial plasmid are not tolerated in the same host, presumably due to incompatibility issues ^7, 9^.

The most efficient and straightforward way to construct knock-out mutants is by transposon mutagenesis ^10^. Transposon mutagenesis is a powerful technique that allows random insertion of DNA into the bacterial chromosome ^11^. Our ultimate aim is to construct a saturation gene knock out library which would be an enormously useful resource for the *Chlamydia* research community. This will enable the accurate identification of all genes essential to chlamydial growth/infectivity – namely, those genes absent from the library, as mutants with insertions in essential genes will not survive being passaged.

In other bacterial species library generation has been routinely achieved by combining purified transposase with a vector carrying the transposon and then transforming the target host with this mixture ^12, 13^. Transposition occurs, and the transposase would then be diluted away. Given the very low frequency of transformation this approach is not feasible in *Chlamydia*. As an alternative, transposon mutagenesis has recently been achieved albeit with a non replicating vector ^14, 15^. This was done by transforming with an *E. coli* based vector (incapable of replication in *Chlamydia*) carrying the *Himar1* C9 transposase under control of a chlamydial promoter and a modified transposon bearing the RSGFP-cat fusion. Whilst transposon mutants were obtained, this was only possible at very low frequency and required multiple transformation experiments and painstaking selection of mutants. This approach is not suitable or efficient for generating mutant libraries at scale.

Our aim was to develop a self-replicating chlamydial vector carrying both the transposon and the transposase under tightly-regulated inducible control so the vector can be used to generate many different libraries under diverse conditons thereby allowing analysis of essential gene functions.

## Methods

### Ethics statement

All genetic manipulations and containment work was approved under the UK Health and Safety Executive Genetically Modified Organisms (contained use) regulations, notification number GM57, 10.1 entitled ‘Genetic transformation of Chlamydiae’.

### Study design and setting

Our study was designed to construct a self-replicating chlamydial vector carrying a transposon and transposase under tightly-regulated inducible control. This work was conducted from March 2017 to February 2020 at the Chlamydia Research Laboratory, Southampton General Hospital.

### Cell culture and chlamydia infection

We selected the strain *C. trachomatis* L2P- ^16^ for these studies, which is a naturally occurring plasmid free strain that we have used as a plasmid recipient in multiple studies. McCoy cells (NCTC, Public Health England, UK) were grown in DMEM supplemented with 10% foetal calf serum. Cells were infected with EBs by centrifugation at 754xg (Beckman Coulter Allegra X-15R centrifuge) for 30 minutes at room temperature in T _25_ tissue culture flasks or 96 well trays for infectivity assays. The infected cells were cultured with medium containing cycloheximide (1μg/ml) and gentamicin (20μg/ml) and incubated at 37°C in 5% CO _2_.

Stocks of *C. trachomatis* L2P- were prepared as above and titres were determined as described below. *C. trachomatis* and McCoy cells were routinely tested for mycoplasma contamination by fluorescence microscopy and using the Lookout Mycoplasma PCR detection kit (Sigma, UK).

### Infectivity assay using X-gal staining

The infectivity assay has been described in detail by Skilton *et al.* ^17^. A mouse monoclonal primary antibody for genus-specific LPS (Chlamydia Biobank Cat. No. #CT601 RRID: AB2721933) was diluted 1:1000 and incubated with methanol-fixed infected cells in 96 well trays for 16h at 4°C. These cells were washed with PBS and incubated with an anti-mouse antibody conjugated with β-galactosidase (Calbiochem) for 1 hour at 37°C. For staining, 100μl of a staining solution [5.0mM K _3_Fe(CN) _6_, 5.0mM K _4_Fe(CN) _6_·3H _2_O, 2.0mM MgCl _2_·6H _2_O, 0.25M 5-bromo-4-chloro-3-indolyl-β-d-galactopyranoside (X-Gal)] was added per well and incubated for 4 hours at 37°C. The chromogenic X-Gal substrate generated blue-stained *C. trachomatis* inclusions, which were then counted and titres were calculated.

### Transformation of *C. trachomatis*

Transformation of *C. trachomatis* L2P- was performed using the calcium chloride based transformation protocol previously described ^7^ with the following minor modification - selection of transformants in T _25_ flasks at passage 1. The experimental procedures were as follows: 3×10 ^7^ IFU of L2P- EBs were pelleted and resuspended in 150μl CaCl _2_ buffer (10 mM Tris pH 7.4 and 50 mM CaCl _2_). 6µg plasmid DNA was diluted in 100μl CaCl _2_ buffer and the two were mixed in a total volume of 250µl, then incubated for 30 mins at room temperature. This mixture was then split equally across 2×T _25_ flasks with 1.5ml/flask of CaCl _2_ and incubated at RT for 20 mins. 5mls/flask of DMEM +10% FCS (with cycloheximide and spectinomycin at 50µg ml ^-1^or penicillin at 10 units ml ^-1^) was then added and the flasks centrifuged at 754xg for 30 mins at RT. The flasks were returned to the incubator for 40 – 48hrs. Infected cells were scraped off the flasks and harvested (as described earlier) into 1ml 10% PBS plus 1ml 4SP for freezing at -80°C. This sample was called T _0_. The selection of transformants was performed by infection of McCoy cells in 1×T _25_ flasks using all of T _0_ (passage 1) and selected with either spectinomycin at 50µg ml ^-1^ or 10 units/ml of penicillin. Two days after infection, the sample was harvested from this flasks as ‘T _1_’. All T _1_ was used to infect McCoy cells in a T _25_ flask with selection relevant to the transforming vector. Passaging was continued in T _25_ flasks with selection until normal inclusions were recovered. The transformants were routinely recovered in passages 2 or 3.

### Microscopy

Cells in culture and cells infected with *C. trachomatis* were routinely visualized by phase contrast microscopy using a Nikon eclipse TS100 inverted microscope with 10x, 20x and 40x objectives and fluorescence accessories. Images were captured using a Nikon DS-Fi1 camera head. Counting of inclusion forming units (IFU) to quantify chlamydial infectivity was performed at 20x magnification on serial dilutions of *C. trachomatis* in monolayers of McCoy cells grown in 96 well trays. For this assay inclusions were immunostained as described in the section “infectivity assay using X-gal staining”.

### *E.coli* strains, plasmid purification and transformation

*E. coli* strain DH5α ^18, 19^ was used for construction of plasmid vectors using standard CaCl _2_ treatment to render the cells competent ^20^. *E. coli* C2925 (NEB) is a Dam-/Dcm- strain, and was used to prepare unmethylated DNA for transformation of *C. trachomatis*. pRPF215 was purchased from Robert Fagan & Neil Fairweather (Addgene plasmid # 106377) ^21^. Plasmids for *C. trachomatis* transformation were purified from *E. coli* C2925 using the Invitrogen midi preparation kit as per the manufacturer’s instructions and assayed for DNA yield by Nanodrop. Plasmids were sequence-verified using the complete plasmid sequencing service using Next-Generation sequencing technology at Massachusetts General Hospital CCIB DNA Core, Cambridge MA, USA. The complete sequence of all the final plasmid constructs are available as FastA files ^22^.

### Immunodetection of C9 transposase by Western blot

Proteins were separated by 12% SDS-PAGE and transferred to a polyvinylidene diflouride (PVDF) Immobilon membrane (EMD Millipore) in Pierce Fast Semi-Dry Buffer (ThermoFisher Scientific) using a Pierce Fast Semi-Dry Blotter. After blocking in 10% skimmed milk/PBS-T solution (Tesco, UK) for 1 hour at room temperature (RT), membranes were incubated with 1:5000 dilution of primary mouse polyclonal antisera to purified C9 transposase ^23^ in 1% skimmed milk/PBS-T for 1 hour at RT. Membranes were washed three times in PBS-T and incubated with secondary antibody (HRP-labelled goat anti-mouse IgG (Bio-Rad)) diluted 1:2000 in 1% skimmed milk/PBS-T for 1 hour at RT. Membranes were finally washed three times and visualised using Pierce enhanced chemiluminescence (ECL) system Western blotting substrate (ThermoFisher Scientific) and developed onto photographic film.

### PCR amplification for cloning experiments

DNA fragments for all cloning experiments were amplified by PCR from prepared plasmid DNA templates. PCR reactions were set up as follows using primer pairs from Table 1; 1x Phusion Flash MasterMix (ThermoScientific), 0.5μM each primer and 1ng plasmid DNA. PCR conditions were 10 seconds at 98°C, followed by 35 cycles of 2 seconds at 98°C, 5 seconds of required annealing temperature of primer pair (see Table 1), and 15 seconds/kb at 72°C, and then a final step of 1 minute at 72°C. PCR was performed using the Veriti 96 Well Thermal Cycler (Applied Biosystems). Products were purified using Promega Wizard SV Gel and PCR Clean-Up System, and concentration determined by NanoDrop 1000 spectrophotometer.

**Table 1.** Primers used in the cloning strategies of the *E.coli/C. trachomatis* shuttle plasmids described in Figure 1, Figure 4 and Figure 6.

Primer Number	Primer Name	Sequence 5’-3’	Restriction Site	Annealing Temp	Target Plasmid	Function
**1** **2**	Erm_del_F Erm_del_R	AAAAAA **GCGGCCGC**CACACTCTTAAGTTTGCTTCTGTC AAAAAA **CTCGAG**GGAGGAAATAATTCTATGAGTCGC	NotI XhoI	52°C	pRPF215	To delete *erm* gene
**3** **4**	Bla_F Bla_R2	AAAAAA **CTCGAG**GAGTAAACTTGGTCTGACAGT AAAAAA **GCGGCCGC**TGGTTTCTTAGACGTCAGGTGGCA	XhoI NotI	52°C	pGFP::SW2	To amplify *bla* gene
**5** **6**	Divergent_F1 Divergent_R1	GGTGGT **GGCCGGCC**AACCTCCTAGTATTATTGAGC GGTGGT **ACGCGT**GGCCTCGGATCCTATAAG	FseI MluI	58°C	pRPF215-Bla	To delete *Tpase(wt)* gene
**7** **8**	C9_F1 C9_R1	GGTGGT **GGCCGGCC**ATGGAAAAAAAGGAATTTCGTG GGTGGT **ACGCGT**TTATTCAACATAGTTCCCTTCAAG	FseI MluI	58°C	pMALC9	To amplify *Tpase(C9)* gene
**9** **10**	Transposon_SalI_F2 Transposon_SalI_R2	GGTGGT **GTCGAC**CACCTCCTTTTTGACTTTA GGTGGT **GTCGAC**GTCCCCATGCGCTCCATCAA	SalI SalI	54°C	pRPF215-Bla- C9Tpase	To amplify tetR/transposon-C9 transposase unit
**11** **12**	Transposon_Deletion_F1 Transposon_Deletion_R1	ATATAT **CGCCGGCG**GCATCTTTTTATTTAGGGATTTCTCAC ATATAT **CGCCGGCG**GTTACCAGTGTGCTGGAATTC	MreI MreI	56°C	pSW2-mCh-C9	To delete transposon unit
**13** **14**	BreakingC9_F1 BreakingC9_R1	ATATAT **CGCCGGCG**GGTGTATTACGAGTTAACAGCTGC ATATAT **CGCCGGCG**GTTCTCAGACCTCAAAAGGATGC	MreI MreI	56°C	pSW2-mCh-C9	To delete active site of *Tpase(C9)* gene
**15** **16**	Short_Transposon_F Transposon_R2	GGTGGT **TCCGGGA**GGCTTCTCTCATGAGAAGTC TTTTTT **TCCGGGA**GTCCCCATGCGCTCCATCAA	PfoI PfoI	58°C	pRPF215-Bla	To amplify transposon unit

## Results and discussion

### 1. Design, construction and testing of a replication-proficient chlamydial transposon delivery vector

It has previously been demonstrated that a basic *E. coli* cloning plasmid (incapable of replication in *Chlamydia*) can be used to deliver an active transposase/transposon system to both *C. trachomatis* and *C. muridarum* ^14, 15^. These studies used the C9 *Himar1* (horn fly) transposase ^24^ coupled with custom-built transposons designed to allow selection of mutants resistant to penicillin ^14^ or chloramphenicol and expressing the green fluorescent protein ^15^. However, due to the very low transformation frequency and the absence of an optimisation protocol for transposase induction, selection of the mutants was a time consuming process. Nevertheless, it provided proof-of-principle that the *Himar1* system functioned in *Chlamydia*.

Here, our overall goal was to develop a self-replicating transposon delivery vector that can be transformed into *C. trachomatis* and then expanded prior to transposase induction at a controlled time point. This would circumvent the severe restriction imposed by low chlamydial transformation frequencies and ultimately make it possible, once we obtained a high titre of transformants, to generate a large pool of mutants.

Our experimental design was to build a C9 *Himar1* transposase/transposon unit organised contiguously on the same plasmid in *cis*. In this construct we engineered the *tet* promoter to regulate transposase expression so that it could be induced at different times during chlamydial development. The *tet* promoter system has previously been shown to be active and its regulatory function operational in *C. trachomatis,* indeed ** this is the only ‘foreign’ inducible promoter system to date that has been identified as functional in *C. trachomatis* ^25^.

**Figure 1.**
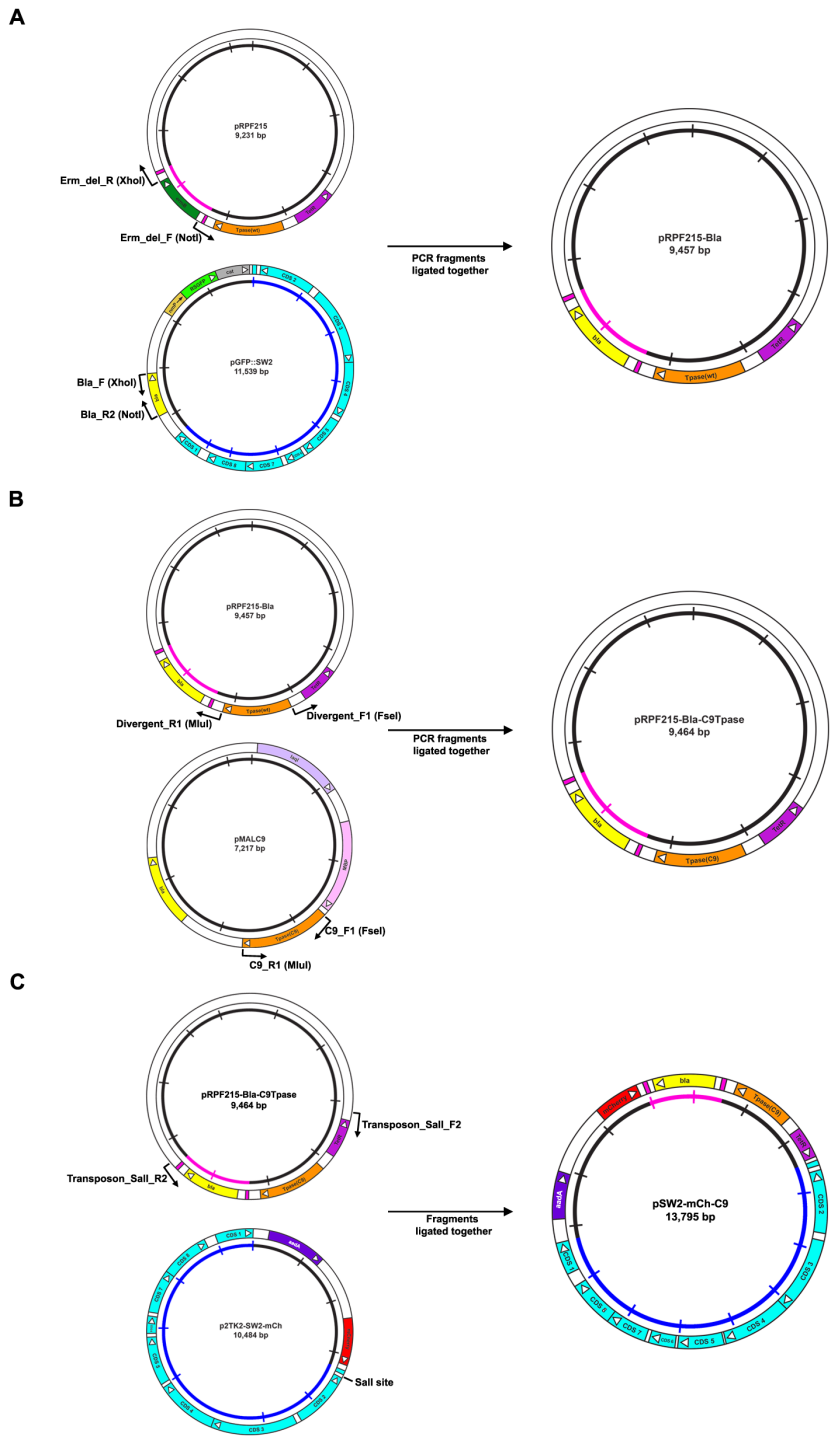
Cloning strategy for the construction of the *C. trachomatis/E. coli* transposon-transposase shuttle vector pSW2-mCh-C9. **A**: Replacement of the erythromycin resistance ( *erm*) gene within the transposon unit with the β-lactamase ( *bla*) gene. The starting point is plasmid pRFP215 ^21^ which carries *erm* as a marker within the transposon. Since erythromycin is used to treat chlamydial infections use of this marker is not permissible under our genetic manipulations licence GM157. The *erm* gene was ‘excised’ by PCR using primers 1 and 2 ( Table 1). The β-lactamase ( *bla*) gene was amplified from our chlamydial shuttle vector pGFP::SW2 ^7^ using primers 3 and 4 ( Table 1). These fragments were ligated to construct plasmid pRPF215-Bla. **B**: Replacement of the *Clostridia* codon-optimised transposase gene with the C9 transposase gene. The original transposase gene which had been codon-optimised for *Clostridia* located in pRPF215-Bla was removed by PCR with primers 5 and 6 ( Table 1). The C9 transposase gene was amplified by PCR with primers 7 and 8 ( Table 1) using plasmid pMALC9 as template. These PCR fragments were ligated to form plasmid pRPF215-Bla-C9Tpase. **C**: Construction of the *C. trachomatis/E. coli* shuttle vector carrying the C9 transposase-transposon. Primers 9 and 10 ( Table 1) were used to amplify the C9 transposase-transposon unit along with its regulatory control region ( *TetR*) from plasmid pRPF215-Bla-C9Tpase. These primers introduce a *SalI* restriction site at both ends of the PCR product. This was then cloned into p2TK2-SW2-mCh (kindly provided by Dr Isabelle Derre) via its unique *SalI* site to generate plasmid pSW2-mCh-C9.

**Figure 2.**
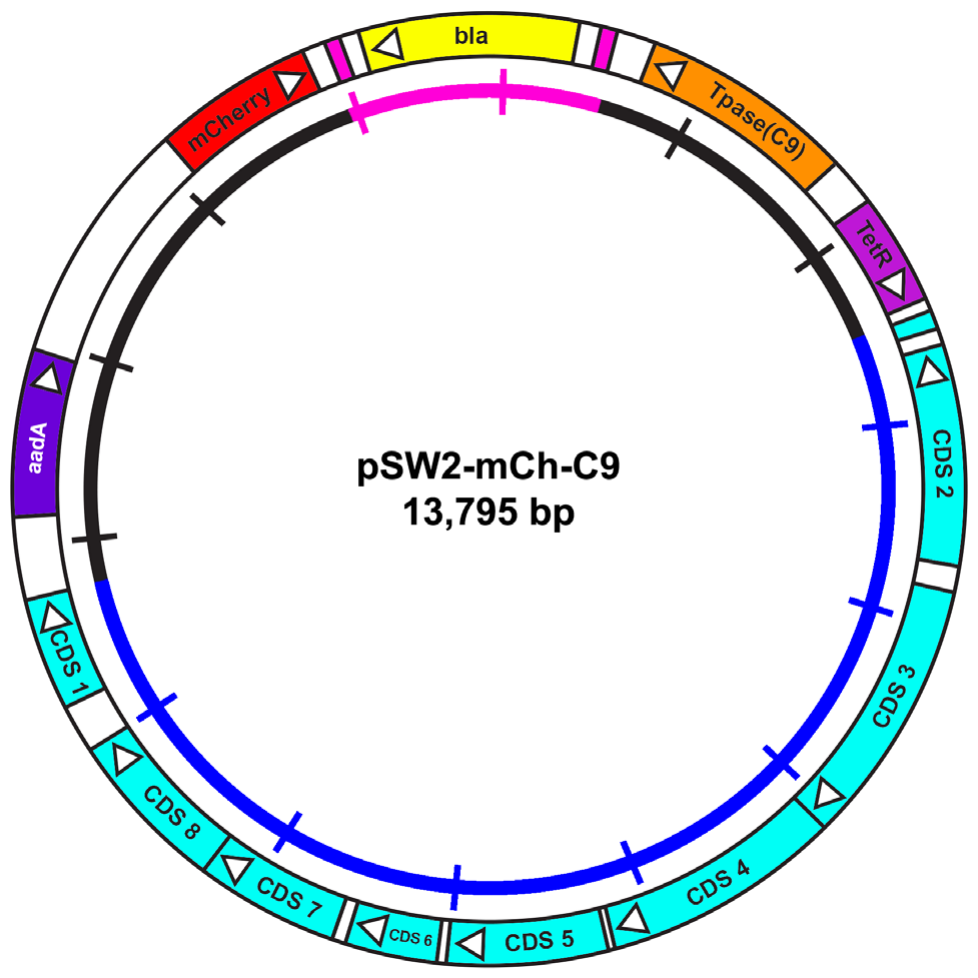
Plasmid map of the *C. trachomatis/E. coli* transposon-transposase shuttle vector pSW2-mCh-C9 showing the essential features for plasmid selection and transposition. The key coding sequences (CDS) and their direction of transcription are represented by the boxes and arrows in the outer circle. The spectinomycin resistance gene ( *aadA*) for selection of plasmid transformants is coloured purple. The β-lactamase gene ( *bla*) is yellow and flanked by the repeat sequences defining the limits of the transposon (pink). The C9 transposase gene (orange) is under inducible control of the *tet* promoter: the tetracycline repressor gene ( *TetR*) is coloured magenta. *E. coli* transformants bearing this plasmid fluoresce red under UV illumination due to the constitutive expression of mCherry (red). The inner circle is a scale bar with 1kb increments, chlamydial plasmid sequences are blue, vector sequences are black and the transposon unit is pink.

The *tet* promoter was included in the design to allow finely tuned transposase induction/expression using the soluble inducer anhydrotetracycline (ATc). The whole functional unit ( *tet* promoter/transposase/transposon) was constructed using existing biologically active vector systems derived from well-studied alternate systems and it was cloned into the chlamydial shuttle vector p2TK2-SW2-mCh ^26^ to give plasmid pSW2-mCh-C9. The cloning strategy and final vector maps are shown in Figure 1 and Figure 2.

**Figure 3.**
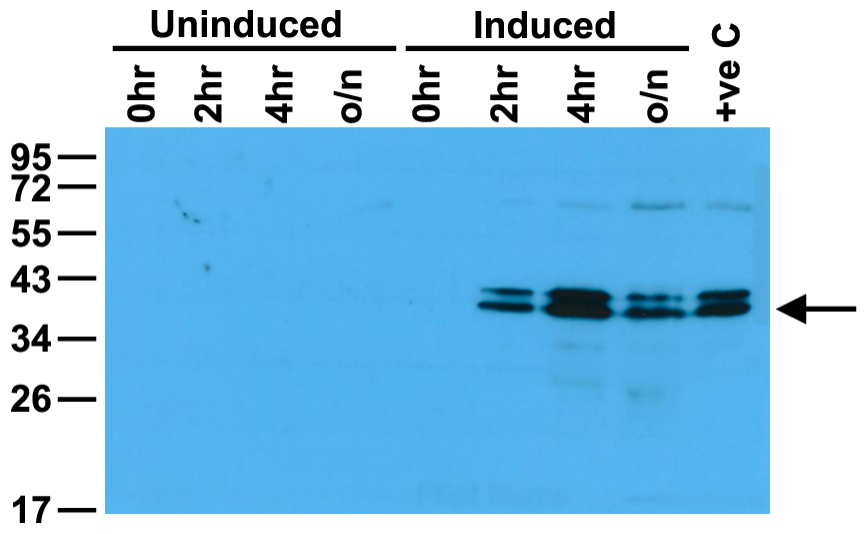
Western blot showing the induction of the C9 transposase in *E. coli*. Equal amounts of protein were loaded in each gel lane. *E. coli* DH5α containing the pSW2-mCh-C9 shuttle vector were grown in liquid culture and induced with ATc and samples taken at the timepoints indicated. Samples were separated by 12% SDS-PAGE gel and the presence of the C9 transposase (arrowed at 40.7kDa) was detected by Western blot with polyclonal antisera to purified C9 transposase.

Plasmid pSW2-mCh-C9 was transformed into *E. coli* strain DH5α. The clones were stable and inducible expression of the C9 transposase was confirmed by Western blot ( Figure 3). This plasmid was then transformed into dam-/dcm- *E. coli* strain C2925 and plasmid DNA prepared for transformation of *C. trachomatis* L2P-. Despite nine separate transformation attempts, with standard chlamydial shuttle vector p2TK2-SW2-mCh as a control (which worked each time) it was not possible to recover transformants with the transposase/transposon bearing plasmid pSW2-mCh-C9 using spectinomycin selection.

These results were confounding and unexpected since plasmid pSW2-mCh-C9 was stable in *E. coli* and the complete absence of any transformants in *C. trachomatis* strongly suggests the pSW2-mCh-C9 shuttle vector is inherently unstable (once it is transformed into *C. trachomatis*) *.*

The chlamydial transformation process requires initial addition of the transforming plasmid DNA to EBs in the presence of calcium chloride and then infection of host cells and the application of selection in subsequent rounds of infection. The routine success of transformation is confirmed by the detection of inclusions by fluorescence microscopy - with mCherry as a marker, transformed EBs will give rise to inclusions that fluoresce red. These are never seen in the first round (T _0_) and thus it is not possible to give a transformation frequency to the process. Several passages are required to amplify a transformed chlamydium before inclusions reach a threshold where they are easily detected. The most likely explanation for the inability to select pSW2-mCh-C9 in *C. trachomatis* is that transposition is occurring from the vector, causing its loss early in the process.

### 2. Uncoupling of the transposon/transposase unit by functional deletion allows recovery of the individual transposon- and transposase-bearing plasmids in *C. trachomatis*

Chlamydial transformation frequencies are so low (possibly only 1–5 transformants per microgramme) therefore, at the very first stage in the process only a single chlamydium will be transformed by a single molecule of the shuttle vector. This transformed bacterium develops into an RB and under selection this will grow and form an inclusion. Several passages under selection are required to amplify that transformant and subsequent single inclusion to a point where it is visually detectable. If transposition occurs due to leaky low level induction of the transposase when there is just one plasmid molecule within an RB, this will lead to excision of the *Himar1* transposon and loss of the delivery vector. This is because active transposase leaves a double stranded DNA break in the donor plasmid when the transposon is excised so the plasmid cannot replicate, causing its loss early in the process ^27, 28^. This will be especially so in the *cis* configuration with both transposase and transposon on the same delivery vector. 

Whilst the *tet* promoter is highly repressed in *E. coli,* and currently is the best choice for finely controlling genes in *Chlamydia*, it seems that very low basal expression of the transposase may occur (in *Chlamydia*) even in the absence of induction (below the level of detection by Western blot) due to very slight leakiness of the *tet* promoter ^25^. 

To test this notion we made ‘functional’ deletions in the pSW2-mCh-C9 plasmid.

***2(a). A deletion mutant crippled for active transposase activity***

**Figure 4.**
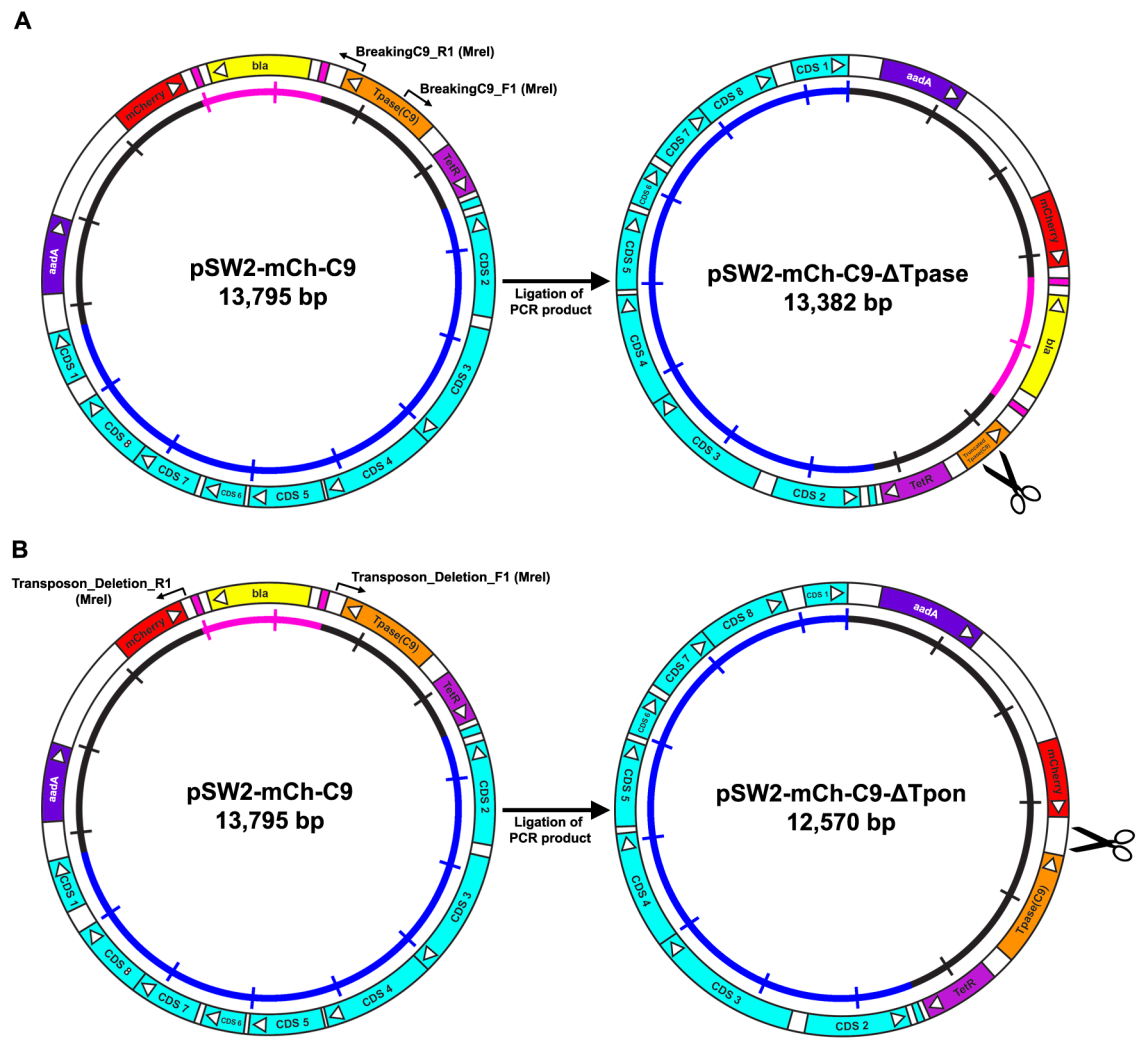
Cloning strategy for the construction of the *C. trachomatis/E. coli* individual transposon and transposase shuttle vectors pSW2-mCh-C9-ΔTpase and pSW2-mCh-C9-ΔTpon. **A**: Deleting the active site of the C9 transposase from pSW2-mCh-C9. The active site of the C9 transposase in pSW2-mCh-C9 was deleted by PCR using primers 13 and 14 ( Table 1), adding *MreI* sites to both ends. The resulting PCR product was re-ligated to form pSW2-mCh-C9-ΔTpase. This deletion (426 bp) is designed to inactivate the transposase generating a truncated protein of predicted molecular weight 17.4kDa ( Figure 5C). The scissor symbol indicates the location of the deletion. **B**: Deletion of the transposon unit from pSW2-mCh-C9. The transposon unit was deleted from pSW2-mCh-C9 by PCR using primers 11 and 12 ( Table 1), adding *MreI* sites to both ends. The resulting PCR product was re-ligated to form pSW2-mCh-C9-ΔTpon. The scissor symbol indicates the location of the deletion.

**Figure 5.**
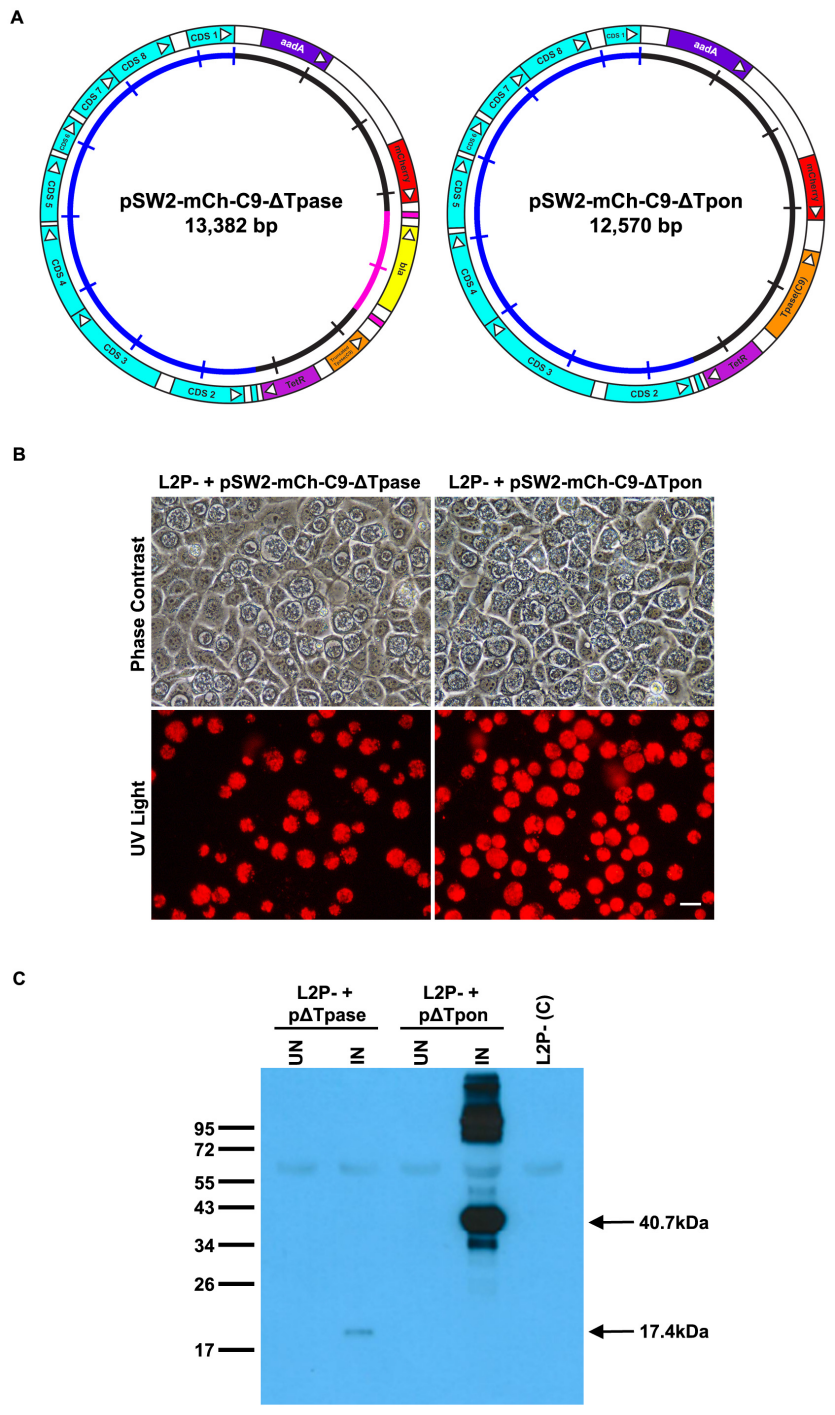
Design and functional analysis of the individual transposon and transposase vectors. **A**: Plasmid maps showing the individual *C. trachomatis/E. coli* shuttle vectors pSW2-mCh-C9-ΔTpase and pSW2-mCh-C9-ΔTpon carrying either the transposon or the C9 transposase respectively. The shared sequence coloured in the outer circle shows the key CDS for chlamydial plasmid replication and maintenance (blue), spectinomycin selection (purple) and the mCherry marker (red) for visualisation of inclusions. In pSW2-mCh-C9-ΔTpase the active site of the C9 transposase has been deleted, in pSW2-mCh-C9-ΔTpon the entire transposon unit has been deleted. The inner circle is a scale with 1kb increments, the transposon sequence in this circle is coloured pink. **B**: Red fluorescent inclusions in McCoy cells infected by *C. trachomatis* L2P- transformed with either pSW2-mCh-C9-ΔTpase or pSW2-mCh-C9-ΔTpon vectors and grown under spectinomycin selection. Phase contrast and UV light images are from the same field at 48h.p.i. Left-hand panels are from L2P- containing pSW2-mCh-C9-ΔTpase and right-hand panels are from L2P- containing pSW2-mCh-C9-ΔTpon. Scale bar represents 20μm. **C**: Western Blot showing the induction on the C9 transposase in *C. trachomatis* L2P- transformed with either pSW2-mCh-C9-ΔTpase or pSW2-mCh-C9-ΔTpon vectors. Equal amounts of protein loaded in each gel lane. McCoy cells were infected with *C. trachomatis* L2P- transformed with either the pSW2-mCh-C9-ΔTpase or pSW2-mCh-C9-ΔTpon vectors. They were induced with ATc at 24h.p.i. and harvested at 48h.p.i. Non-induced and un-transformed L2P- were used as controls. Samples were separated by 12% SDS-PAGE gel and the presence of the truncated C9 transposase (arrowed at 17.4kDa) or C9 transposase (arrowed at 40.7kDa) was detected by Western blot with polyclonal antisera to purified C9 transposase.

If the expression of the C9 transposase in the presence of its target functional transposon is the cause of pSW2-mCh-C9 instability/loss in *C. trachomatis* then a simple inactivating mutation in the transposase should allow recovery of the deleted construct. Therefore, a deletion of 426 bp was made of the *Himar1* C9 transposase gene of pSW2-mCh-C9 by PCR. This deletion removed the whole of the catalytic domain of the C9 transposase causing it to be prematurely truncated when expressed. The cloning strategy is shown in Figure 4A and the final vector map for pSW2-mCh-C9-ΔTpase is shown in Figure 5A. In stark contrast to pSW2-mCh-C9, the deleted transposase version was routinely and easily recovered by transformation of *C. trachomatis* L2P- and selection with spectinomycin ( Figure 5B). Western blots of transformed L2P- carrying pSW2-mCh-C9-ΔTpase induced with ATc showed induction of the truncated transposase ( Figure 5C). These data confirmed that our inability to recover pSW2-mCh-C9 in *C. trachomatis* is due to the presence of the active C9 transposase in that vector since all other parts of the plasmid pSW2-mCh-C9-ΔTpase remain identical to pSW2-mCh-C9.

***2(b). Inactivation of the transposon***

If our hypothesis about the active C9 transposase in the context of the transposase/transposon combination on plasmid pSW2-mCh-C9 is correct then inactivation of the transposon will also allow recovery of stably transformed *C. trachomatis* able to carry (and express) the complete transposase. Deletion of the whole transposon unit (repeat sequences and *bla* gene) was performed by PCR (shown in Figure 4B) and the resulting plasmid, pSW2-mCh-C9-ΔTpon ( Figure 5A), was used to transform *C. trachomatis* L2P-. These transformants were stable and easily expandable ( Figure 5B). Following induction with ATc, Western blotting showed that the complete C9 transposase was both inducible and expressed well in *C. trachomatis* ( Figure 5C).

### 3. Lateral gene transfer of the transposon

We have demonstrated that it is possible to recover two independent stable transformants in *Chlamydia*, one able to carry the transposon and a second able to express the transposase under inducible control (apart from the specific deletions both these plasmids share identical backbones). These observations are consistent with our working hypothesis that it is not possible to recover the combined transposase/transposon plasmid because there is low level leakage/basal expression of the transposase in *C. trachomatis* from the *tet* promoter.

In bacterial systems with no, or extremely low-frequency genetic transformation systems, an alternate way of introducing transposon plasmids is by conjugation ^29, 30^. Unfortunately, there is no conjugative system for *Chlamydia*. However, it is possible to exchange DNA between *Chlamydia* either within the same species ^31^ and also between different species ^32^ by co-infection, a process known as lateral gene transfer. The mechanism(s) that allow *Chlamydia* to exchange DNA in the lateral gene transfer process have not been characterised. Co-infection of cells by two strains, each carrying a different chromosomally-located antibiotic-selectable marker allows recovery of hybrid progeny following dual detection; these ‘offspring’ have undergone chromosomal recombination and the progeny carry both selectable markers on the same chromosome. We sought to investigate whether we could introduce the individual transposase and transposon bearing plasmids into the same RBs by mixed infection and dual selection. As a first step, we needed to construct a second vector with different fluorescent markers and different selectable genes - these were needed to monitor for mixed infection and then to select for progeny carrying both plasmids.

***3(a). Functional verification of engineered markers***

**Figure 6.**
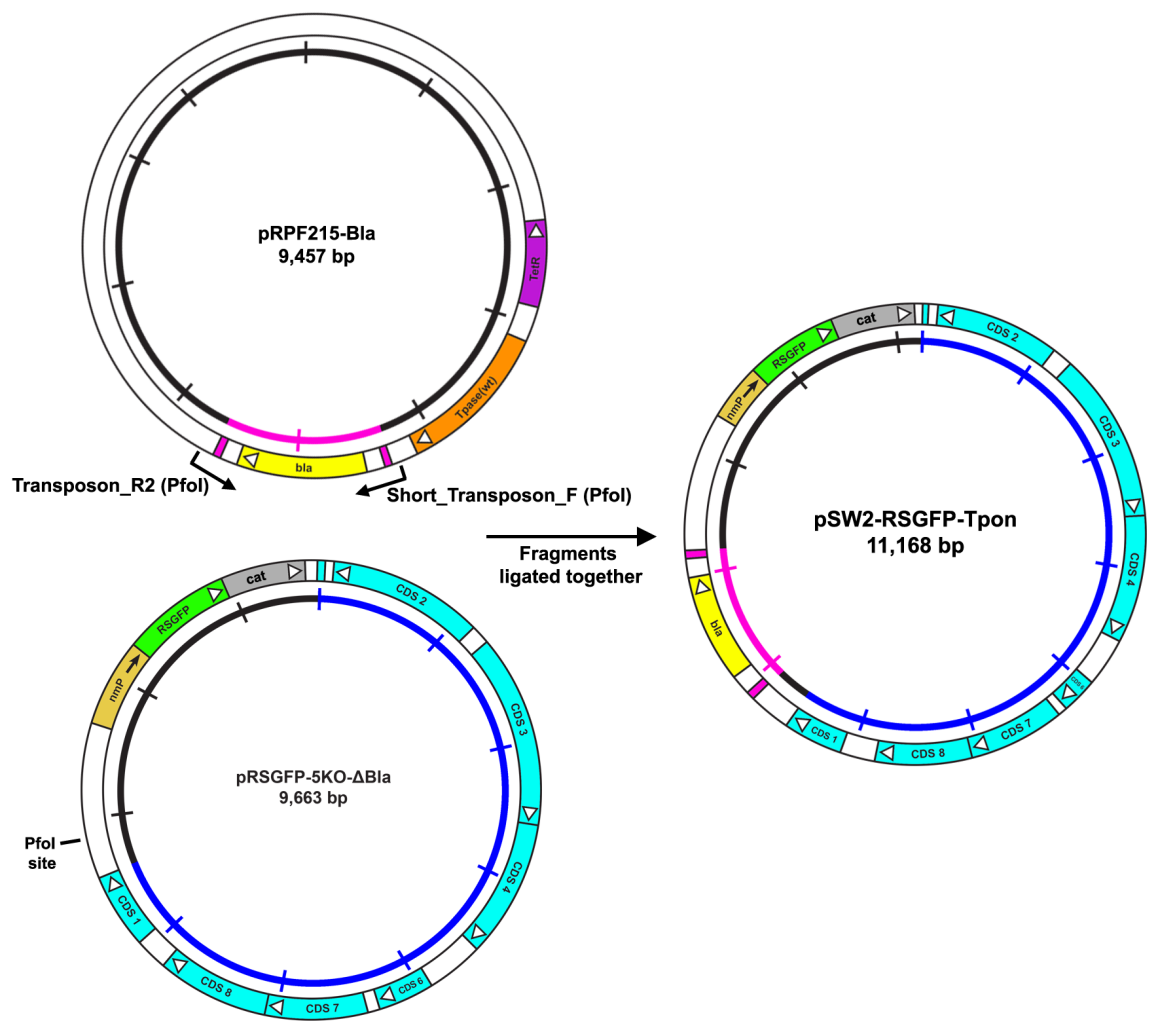
Cloning strategy for the construction of the *C. trachomatis/E. coli* transposon shuttle vector pSW2-RSGFP-Tpon. Primers 15 and 16 ( Table 1) were used to amplify the transposon unit carrying the β-lactamase gene (yellow) from plasmid pRPF215-Bla. These primers introduce a *PfoI* restriction site at both ends of the PCR product. This was then cloned into pRSGFP-5KO-ΔBla via its unique *PfoI* site to generate plasmid pSW2-RSGFP-Tpon.

**Figure 7.**
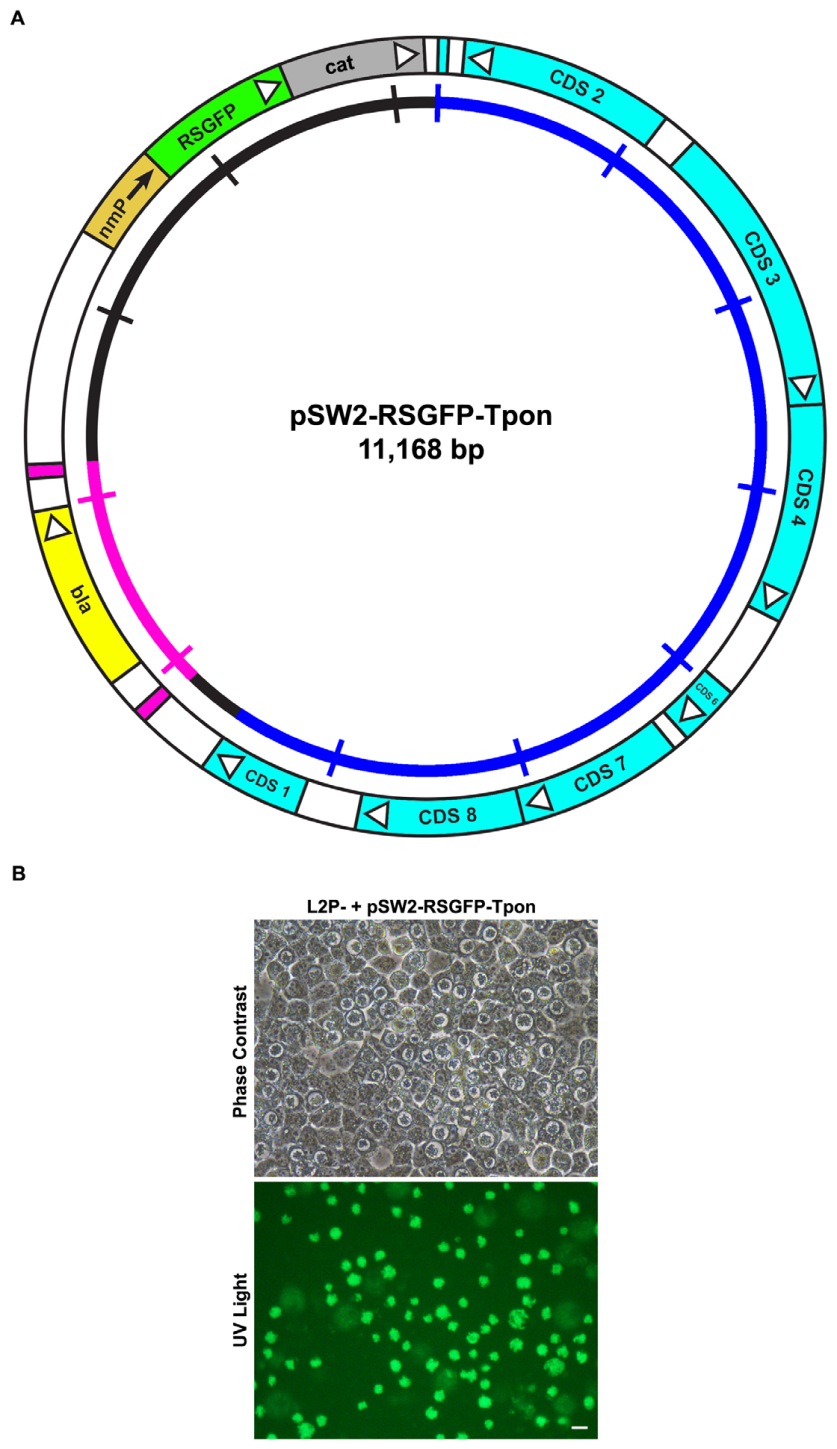
Design and functional analysis of the green version of the transposon shuttle vector. **A**: Plasmid map of the *C. trachomatis/E. coli* transposon shuttle vector pSW2-RSGFP-Tpon showing the essential features for plasmid selection and transposition. The key CDS and their direction of transcription are represented by the boxes and arrows in the outer circle. The β-lactamase gene ( *bla*) is yellow and flanked by the repeat sequences defining the limits of the transposon (pink). *E. coli* transformants bearing this plasmid fluoresce green under UV illumination due to the constitutive expression of RSGFP (green). The inner circle is a scale bar with 1kb increments, chlamydial plasmid sequences are blue, vector sequences are black and the transposon unit is pink. **B**: Green fluorescent inclusions in McCoy cells infected by *C. trachomatis* L2P- transformed with pSW2-RSGFP-Tpon vector and grown under penicillin selection. The top panel shows McCoy cells infected at 48h.p.i. under phase contrast. The lower panel is of the same field under UV light illumination. The scale bar represents 20μm.

Our experimental design was to use one of our existing chlamydial transformants as a donor for the mixed infection. Since both our constructs (pSW2-mCh-C9-ΔTpon and pSW2-mCh-C9-ΔTpase) were in the same plasmid backbone with the same selectable and fluorescent markers (both display red fluorescence from expression of the mCherry gene), one had to be redesigned. We chose the transformant bearing plasmid pSW2-mCh-C9-ΔTpon as parent 1 ( Figure 5A) since we have already demonstrated that the transposase is inducible, this strain is spectinomycin resistant and carries the mCherry marker. We designed plasmid pSW2-RSGFP-Tpon as parent 2. The cloning strategy is shown in Figure 6. Briefly, it is based on one of our existing cloning vector constructs for *C. trachomatis* which has ORF 5 deleted (a plasmid CDS not required for maintenance) to facilitate maximal insertion size, and also to enable differentiation of backbones in the event of recombination ^33^. The original shuttle plasmid carries the β-lactamase gene and separately a green fluorescence protein (RSGFP). RSGFP is contiguous with the chloramphenicol acetyl transferase gene and both expressed from a constitutive neisserial promoter. This allows selection of transformants by the presence of green inclusions that are resistant to chloramphenicol and/or penicillin. To avoid complications from gene duplication (the transposon carries an identical *bla* gene) the *bla* gene on the shuttle vector was deleted and plasmid pRSGFP-5KO-ΔBla was used as recipient of the *bla* gene within the transposon unit. Final vector map is shown in Figure 7A.

*E. coli* transformed by pSW2-RSGFP-Tpon was chloramphenicol and ampicillin resistant and fluoresced green under UV light compared with *E. coli* transformed with pSW2-mCh-C9-ΔTpon which was spectinomycin resistant only and fluoresced red. 

A deficiency in the verification of the plasmid functions was that whilst we had shown the transposase was inducible (by Western blot with hyper immune sera), we had not shown that it was functional. Before we embarked on a complex series of genetic experiments in *C. trachomatis* it was necessary to demonstrate transposition in *E. coli* carrying both pSW2-mCh-C9-ΔTpon and pSW2-RSGFP-Tpon. We made competent cells of *E. coli* carrying pSW2-mCh-C9-ΔTpon - these cells were divided into two sets at exponential phase, one with ATc (induced) and one without (uninduced). Both sets were then transformed with pSW2-RSGFP-Tpon and tranformants selected on ampicillin plates. Transposon progeny (where the transposon has jumped from pSW2-RSGFP-Tpon to *E. coli* carrying pSW2-mCh-C9-ΔTpon) will be ampicillin resistant and fluoresce red. The parental pSW2-mCh-C9-ΔTpon strain will not grow under this selection and *E. coli* carrying pSW2-RSGFP-Tpon yields ‘green’ ampicillin resistant colonies.

**Figure 8.**
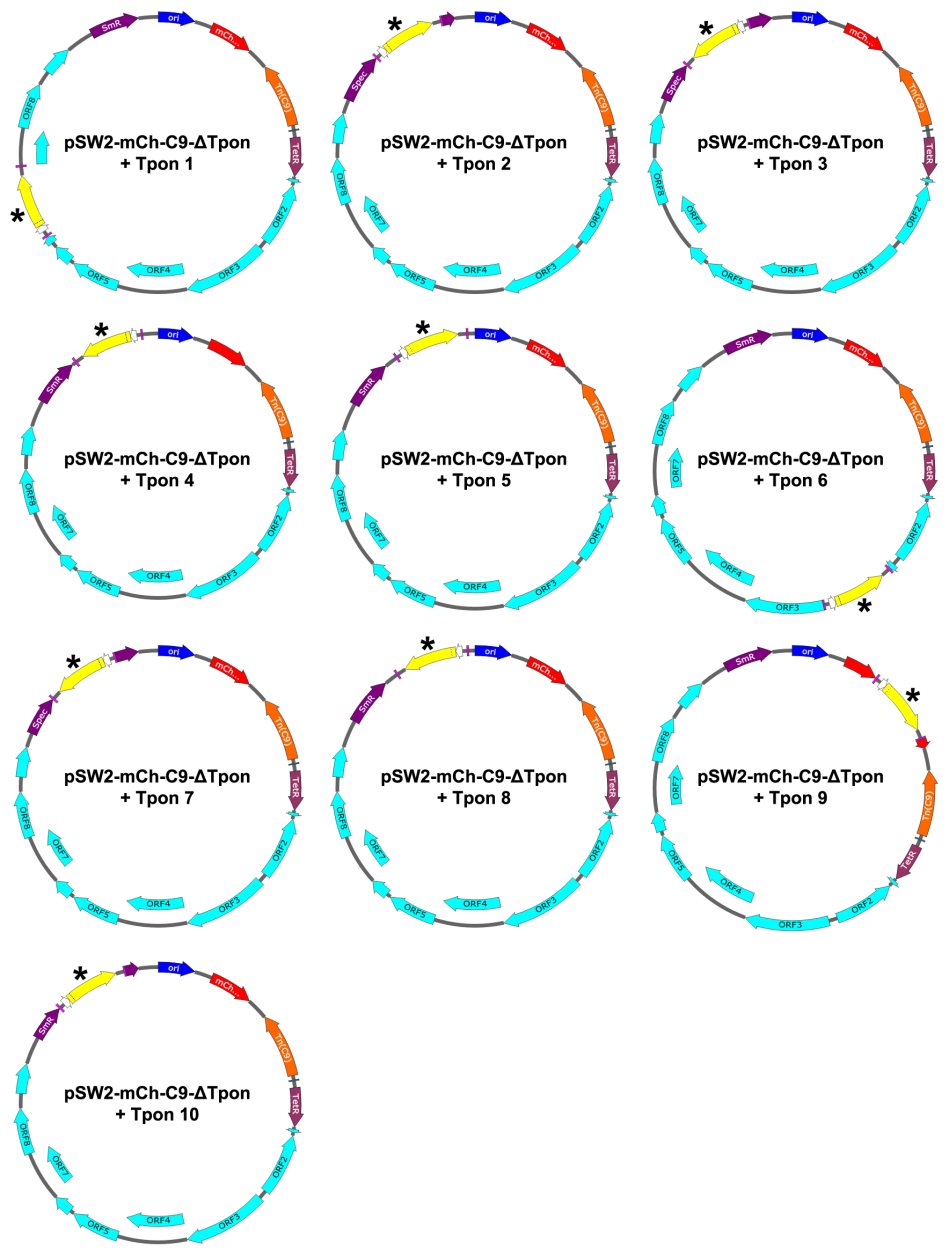
Plasmid maps of the pSW2-mCh-C9-ΔTpon vectors with transposon insertions. Plasmid maps showing the location of the integrated transposon units that were transposed from pSW2-RSGFP-Tpon into the pSW2-mCh-C9-ΔTpon plasmid giving rise to red, ampicillin resistance in *E. coli* colonies. Maps drawn from sequence data using SnapGene. The inserted ampicillin gene is coloured yellow and highlighted with an asterisk. Plasmid maps are orientated by the *E. coli* replication origin (dark blue). Plasmid sequences available as FastA files ^22^.

Transformation of *E. coli* carrying pSW2-mCh-C9-ΔTpon with 10ng pSW2-RSGFP-Tpon gave approximately a thousand green colonies from both tranformations (with and without ATc induction) on ampicillin plates as expected, whereas red colonies (indicating transposition) were only recovered from the induced competent cells. A selection of these were used to make plasmid DNA which was re-transformed into *E. coli* to select for individual colonies that fluoresced red. Plasmid DNA from 10 individual colonies was purified and sequenced ( Figure 8). This showed transposition between pSW2-RSGFP-Tpon and pSW2-mCh-C9-ΔTpon in *E. coli* and confirmed the transposase was active.

***3(b). Attempts at lateral gene transfer of pSW2-mCh-C9-ΔTpon and pSW2-RSGFP-Tpon by co-infection in *C. trachomatis****

**Figure 9.**
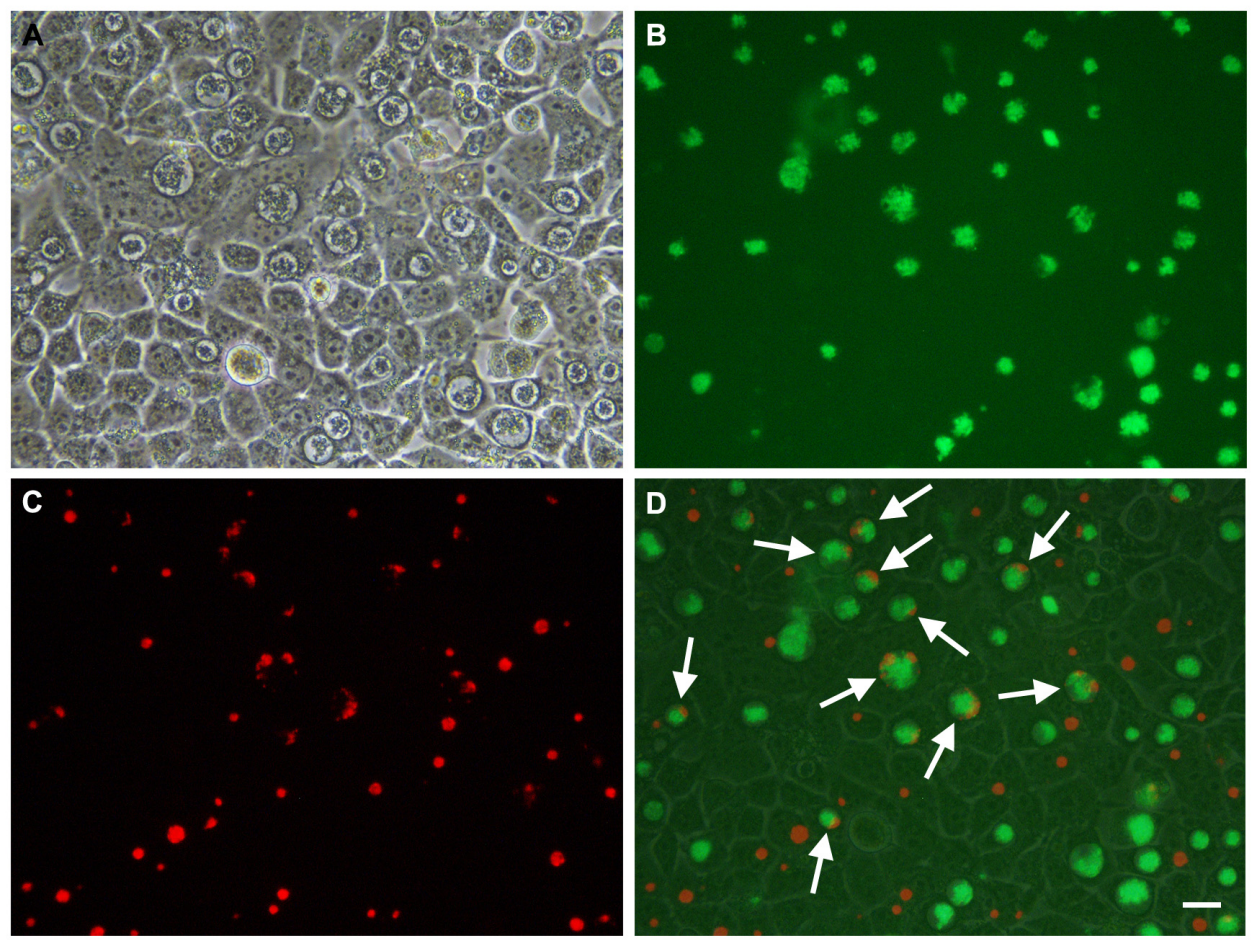
Mixed infection of McCoy cells infected with *C. trachomatis* L2P- transformed with pSW2-RSGFP-Tpon vector and *C. trachomatis* L2P- transformed with pSW2-mCh-C9-ΔTpon vector, grown with no selection. This figure shows an example of the same microscopic field observed under phase contrast (Panel **A**), UV light (green filter) (Panel **B**) and UV light (red filter) (Panel **C**). Panel **D** is a merged image of the phase, green (L2P- transformed with pSW2-RSGFP-Tpon) and red (L2P- transformed with pSW2-mCh-C9-ΔTpon). White arrows highlight inclusions showing both green and red fluorescence. Scale bar represents 20μm.

With the biological/ functional activities of all the vectors verified, our aim was to investigate whether we could introduce individual transposase and transposon plasmids into the same chlamydial host by lateral gene transfer, following co-infection. Plasmid pSW2-RSGFP-Tpon was transformed into *C. trachomatis* L2P- and green penicillin/chloramphenicol resistant inclusions were obtained, these were stable and grew well ( Figure 7B). Cells were then co-infected with *C. trachomatis* L2P- containing pSW2-mCh-C9-ΔTpon and pSW2-RSGFP-Tpon at MOI=1.0 (this was chosen to maximise chance of co-infection yet keeping below the threshold of over infectivity). We were consistently able to obtain mixed infections (as measured by dual fluorescing inclusions) ( Figure 9). Induction with ATc was performed at 24hrs post infection and cultures harvested for analysis at 48hrs.

Despite repeated success in achieving mixed inclusions at optimal frequency, we were not able to select any *Chlamydia* that fluoresced red and were penicillin resistant. Since all the biological components of the vectors were functional, the inability to obtain transposition mutants strongly suggests that stable plasmid transfer/co-existence of pSW2-mCh-C9-ΔTpon and pSW2-RSGFP-Tpon in *C. trachomatis* is not possible. However, it has been possible to force chlamydial plasmid recombination by antibiotic selection following transformation ^9^. Therefore, in a final series of experiments we attempted to mimic the experiments performed with *E. coli* ( section 3 (b)) and tried to transform *C. trachomatis* L2P- carrying the pSW2-mCh-C9-ΔTpon plasmid with pSW2-RSGFP-Tpon but were unable to select any red penicillin resistant transformants. These observations support our hypothesis that it is not possible for the C9 transposase or transposon to exist on a naturally occurring recombinant plasmid in this expression system.

## Conclusions

We have designed and assembled an ‘ideal’ chlamydial vector ‘pSW2-mCh-C9’ for transposition that is stable in *E. coli* but it has not proven possible to recover this vector in *C. trachomatis*. By selective deletion of pSW2-mCh-C9 we have demonstrated that the transposase and transposon are functional and are individually stable in *C. trachomatis*. *C. trachomatis* has only one type of plasmid and so for efficient delivery of transposons the transposase and transposon have to be on the same vector. However, our data suggests that leaky expression of the transposase is responsible for premature loss of the transposon plasmid, thereby preventing establishment of stable clones in *C. trachomatis* carrying this engineered vector configuration (pSW2-mCh-C9). Our previous studies have shown that when two ‘competing’ chlamydial plasmids are present in the same host one is either eliminated or recombination occurs. Therefore, we also attempted both co-infection and transformation to bring the two individually stable plasmids (pSW2-mCh-C9-ΔTpon and pSW2-RSGFP-Tpon) together in the same bacterium in the hope both would co-exist under dual selection and allow transposition. Whilst this approach worked in *E. coli* (by using direct transformation) the process was not a success in *C. trachomatis*.

The options for overcoming low-level basal expression of the transposase are limited. Guaranteeing binary complete on/off control of promoters remains a biotechnological challenge. Our understanding of gene regulation in *Chlamydia* lags far behind other bacterial systems and there are no bespoke chlamydial expression systems with high level regulatory features. We have used the highly regulated *tet* promoter system which is currently the best option for *Chlamydia*. It may be possible to tighten regulatory controls by increasing repressor concentration or operator binding affinity for the repressor protein. Therefore, the solution for repressing transposase expression lies in adding additional control sequences or trying other highly repressed bacterial expression systems that might work in *Chlamydia*. We hope that our results here are informative and will guide the field in finding a way to achieve saturation mutagenesis in *Chlamydia*.

## Data availability

### Underlying data

Open Science Framework: Progress towards an inducible, replication-proficient transposon delivery vector for Chlamydia trachomatis. https://doi.org/10.17605/OSF.IO/5F2PE ^22^.

This project contains the following underlying data:

- The complete sequence of all the final plasmid constructs (FastA files)
- Underlying images for Figures 3, 5, 7, 9

Data are available under the terms of the Creative Commons Zero "No rights reserved" data waiver (CC0 1.0 Public domain dedication).
