# Supplementary material for: Progress towards an inducible, replication-proficient transposon delivery vector for Chlamydia trachomatis
Source: Wellcome Open Res. 2021 Apr 13;6:82. [Version 1] doi: 10.12688/wellcomeopenres.16665.1 (PMC8097735; doi:10.12688/wellcomeopenres.16665.1)
Supplement: Peer review history

# Peer review: Reviewer response for version 1

**DOI:** 10.21956/wellcomeopenres.18376.r43470

**Published:** 2021-05-04

**Authors:** Ted Hackstadt, Zoe Dimond

**Affiliations:** 1 Host-Parasite Interactions Section, Laboratory of Bacteriology, Rocky Mountain Laboratories, NIAID, National Institutes of Health (NIH), Hamilton, MT, USA; 2 Rocky Mountain Labs, National Institute of Allergy and Infectious Disease, National Institutes of Health, Hamilton, MT, USA

*Chlamydia trachomatis* is the causative agent of several diseases of humans including sexually transmitted infections and blinding trachoma. Chlamydiae are bacterial obligate intracellular pathogens that had long been recalcitrant to recombinant DNA technologies. Ten years ago, Wang et al provided the first demonstration of stable transformation of chlamydiae and introduced a decade of remarkable advances. Despite the tremendous progress provided by genetic tools in understanding chlamydial pathogenesis, obstacles to genetic manipulation remain. The absence of a bacteriological medium to axenically cultivate chlamydiae in the absence of host cells, coupled with a developmental cycle in which the infectious form is metabolically downregulated and (relatively) environmentally resistant, presents challenges that are frequently difficult to anticipate or overcome. During the push to develop genetic tools for chlamydiae, there were frequent discussions within the field about the benefits of presenting even negative results provided the experiments were carefully done and informative. The thought being that others need not repeat unproductive approaches and could work toward improvement of those studies. The studies presented here by Skilton *et al. *fit perfectly within the aims of that notion. 

 Transposon mutagenesis is a powerful tool to create libraries of disrupted genes to assess function and can aid in the identification of essential genes. Although transposon mutagenesis of *C. trachomatis* has been reported, the efficiencies remain low enough to limit utility and saturation. In the current report, efforts are made to develop a self-replicating transposon delivery vector for *C. trachomatis* that can be expanded before transposase induction. Plasmids containing both the transposase under tet-promoter control and the transposon could not be recovered from chlamydiae. Plasmids containing the transposase or the transposon could be separately transformed into *C. trachomatis*. Attempts to get both plasmids into single replicating chlamydia were also unsuccessful. Collectively, the negative results were attributed to low levels of transposase expression by the leaky tet-promoter. The conclusions may stimulate a search for a more tightly regulated inducible promoter in *Chlamydia.*

Is the work clearly and accurately presented and does it cite the current literature?

Yes

If applicable, is the statistical analysis and its interpretation appropriate?

Yes

Are all the source data underlying the results available to ensure full reproducibility?

Yes

Is the study design appropriate and is the work technically sound?

Yes

Are the conclusions drawn adequately supported by the results?

Yes

Are sufficient details of methods and analysis provided to allow replication by others?

Yes

Reviewer Expertise:

Biology of Intracellular parasites

We confirm that we have read this submission and believe that we have an appropriate level of expertise to confirm that it is of an acceptable scientific standard.

# Peer review: Reviewer response for version 1

**DOI:** 10.21956/wellcomeopenres.18376.r43469

**Published:** 2021-04-16

**Authors:** Isabelle Derré

**Affiliations:** 1 Department of Microbiology, Immunology, and Cancer Biology, University of Virginia, Charlottesville, VA, USA

*Chlamydia trachomatis* is an important human pathogen responsible for ocular and sexually transmitted infections. In addition to an obligate intracellular life style, the lack of genetic tools has complicated the study of this important human pathogen.

 10 years ago, this group developed a transformation method for *Chlamydia* that revolutionized the field and opened the door to targeted mutagenesis. Recently a transposition system was developed (Wang et al 2019 and LaBrie et al 2019); however, the transposition frequency is low, preventing to reach saturation and establish a large collection of *Chlamydia* mutants.

 In this manuscript Skilton et al set out to attain saturated mutagenesis by transposition in *Chlamydia*. Two approaches are described:

1. A single plasmid carrying the Himar1 C9 transposase under an inducible promoter and the Himar1 transposon.
2.  The use of lateral gene transfer between chlamydia strains each harboring the Himar1 transposon or the Himar1 C9 transposase under an inducible promoter.

Sadly, although these vectors were functional in *E. coli*, attempt at transposition using both approaches failed in *C. trachomatis*. Nevertheless, the authors have generated valuable vectors and there is value in sharing these unsuccessful approaches to allow the field to move forward by modifying rather than repeating these approaches. As noted by the authors, the first step would be to develop a tighter inducible system.

Is the work clearly and accurately presented and does it cite the current literature?

Yes

If applicable, is the statistical analysis and its interpretation appropriate?

Not applicable

Are all the source data underlying the results available to ensure full reproducibility?

Yes

Is the study design appropriate and is the work technically sound?

Yes

Are the conclusions drawn adequately supported by the results?

Yes

Are sufficient details of methods and analysis provided to allow replication by others?

Yes

Reviewer Expertise:

Chlamydia, cell biology and genetics

I confirm that I have read this submission and believe that I have an appropriate level of expertise to confirm that it is of an acceptable scientific standard.
